# Tre2 (USP6NL) promotes colorectal cancer cell proliferation via Wnt/β-catenin pathway

**DOI:** 10.1186/s12935-019-0823-0

**Published:** 2019-04-16

**Authors:** Kang Sun, Song-Bing He, Yi-Zhou Yao, Jian-Guo Qu, Rong Xie, Yu-Qiao Ma, Ming-Hui Zong, Ji-Xiang Chen

**Affiliations:** 1 Department of General Surgery, The Affiliated Hospital of Jiangsu University, Zhenjiang, 212001 People’s Republic of China; 2 Department of General Surgery, The First Affiliated Hospital of Soochow University, Suzhou, 215006 People’s Republic of China; 3 School of Medicine, Jiangsu University, Zhenjiang, 212013 People’s Republic of China

**Keywords:** USP6NL, Colorectal cancers, Wnt/β-catenin pathway

## Abstract

**Background:**

Most colorectal cancer (CRC) patients are diagnosed at an advanced or metastatic stage with poor prognosis. Ubiquitin-specific protease 6 N-terminal-like protein (USP6NL) with high expression in CRC tissues regulates CRC cell proliferation via Wnt/β-catenin pathway. We hypothesized that USP6NL impacts CRC growth and inhibition of USP6NL may be a novel treatment strategy to improve CRC therapy.

**Methods:**

USP6NL level in human CRC tissues and its association with tumor growth and metastasis were examined. Its roles and potential mechanisms in regulating tumor growth were studied by genetic and pharmacological manipulation of CRC cells in vitro and in vivo.

**Results:**

Herein, we found that USP6NL was up-regulated in tumorous tissues of CRC patients. Our data suggested that knockdown of USP6NL in human CRC cell lines (HCT116 and LOVO cells) inhibited cell proliferation, induced G0/G1 cell cycle arrest, and prevented the tumorigenicity of HCT116 cells in nude mice, and which was associated with the prevention of Wnt/β-catenin pathway. On the contrary, USP6NL overexpression in human CRC cells (SW480) showed the opposite result. Our data suggested that the promoted cell proliferation, G1/S cell cycle progression, and the enhanced expression of β-catenin Cyclin D1 and C-myc while reduced P27 induced by the overexpression of USP6NL were significantly reversed by additional treatment of XAV939, indicating that activating Wnt/β-catenin pathway was the mechanism, by which USP6NL exerted carcinogenesis in CRC in vitro. Besides, our data suggested that knockdown of USP6NL increased the ubiquitination of β-catenin, indicating that USP6NL may serve as a deubiquitinase that regulated β-catenin accumulation in this process. Furthermore, 10058-F4 down-regulated USP6NL, inhibited CRC cell proliferation and induced cell cycle arrest. The result demonstrated a possible feedback loop between USP6NL, β-catenin and C-myc in regulating CRC cell growth.

**Conclusion:**

USP6NL was an oncogene in CRC, and it may be a potential target for the treatment of CRC.

## Background

GTPase-activating proteins (GAPs) family proteins can bind to activated G proteins and motivate their GTPase activity, impairing the binding with downstream signaling targets [1]. GAP activity is essential for its oncogenic function [2]. Ubiquitin-specific protease 6 N-terminal-like protein (USP6NL), or RN-tre, is a GTPase-activating protein for Rabs, thereby is involved in control of cell migration, endocytosis of PM receptors and retrograde transport of Golgi [3, 4]. As a member of RabGAPs, USP6NL harbors an TBC (Tre-2/Bub2/Cdc16) domain [4], which exerts oncogenic activity in many cancers [5]. USP6NL is up-regulated and predicts a poor prognosis in human breast cancer (BC) [6]. However, the roles of USP6NL in human colon carcinoma (CRC) remained largely unknown.

Wnt/β-catenin signaling is essential in carcinogenesis, embryonic development and tissue homeostasis [7]. Activating Wnt signaling cascade contributes to the etiology of CRC during intestinal epithelium homeostasis and differentiation [8]. These primarily consist of recruitment of the transcription factor β-catenin in the nucleus and the stimulation many downstream oncogenes, for example c-Myc and Cyclin D-1 [7]. Increased expression of β-catenin has been consistently found in tumorous colon tissue, and predicts poor survival of CRC patients [9]. However, the mechanisms of the upregulation of β-catenin in CRC need to be fully understood.

Ubiquitin-specific proteases (USPs), acting as deubiquitinating enzymes (DUBs), is emerging as master regulator of Wnt/β-catenin signaling [10]. We found that USPs members such as USP4, USP7 and USP47 have been identified as a β-catenin-specific deubiquitinylating enzyme, and involved in the increased stability of β-catenin protein [10–12]. USPs member USP6 positively regulates WNT/β-catenin pathway and facilitates β-catenin nuclear accumulation [13]. Homologous to USP6, USP6NL contains the domain that encodes a deubiquitinating enzyme [14]. However, the mechanism of USP6NL acted on Wnt/β-catenin signaling pathway in CRC remained unclear.

Here, we found that USP6NL was up-regulated and functioned as an oncogene in human CRC cells. USP6NL favored β-catenin accumulation in CRC cells and regulated its downstream oncogenes (P27, Cyclin D1 and C-myc). As a result, the Wnt/β-catenin signal pathway is activated and promoted pro-proliferation and normal cell cycle progression of CRC cells.

## Materials and methods

### Bioinformatics analysis

To study the role of USP6NL in CRC, mRNA expression of USP6NL in 32 pairs of tumorous colorectal tissues and adjacent non-tumorous tissue were assessed, using RT-PCT, in CRC patients recruited at the Affiliated Hospital of Jiangsu University. The researches were supported by the Independent Ethics Committee (IEC) of the Affiliated Hospital of Jiangsu University and all patients were provided written informed consent. Besides, mRNA expression data of USP6NL in 260 tumorous colorectal tissues and 41 none-tumorous adjacent tissues were downloaded from The Cancer Genome Atlas database (TCGA) (https://cancergenome.nih.gov/researchhighlights/researchbriefs/ColorectalCancerInsights). Furthermore, mRNA expression data of USP6NL in 70 colorectal tissues and 12 adjacent tissues were deposited in NCBIs Gene Expression Omnibus (GEO, http://www.ncbi.nlm.nih.gov/geo/) with accessible number GES9348. Immunohistochemistry (IHC) analysis was performed to assess USP6NL expression in 10 tumorous tissues samples and 5 precancerous tissue from patients and with CRC in the Affiliated Hospital of Jiangsu University. The use of CRC patients was permitted by the ethics committee of the Affiliated Hospital of Jiangsu University, and written informed consent of each participate were acquired.

### Immunohistochemistry (IHC) assay

Expression pattern of USP6NL in normal or tumorous colorectal tissues was assessed using IHC method. Colorectal tissue sections (4–7 μm thick) were incubated with USP6NL antibody (ab233414, Abcam) at 4 °C overnight followed by secondary antibodies (D-3004, Long Island Biotech, China) at 25 °C for 30 min. DAB substrate (Long Island Biotech) and hematoxylin (714094, BASO) was used for coloration. A light microscope (Eclipse Ni-E/Ni-U, NIKON) with an image analysis system (DS-Ri2, NIKON) were used for visualization and calculation of USP6NL-positive cell areas.

### Cell culture and treatment

HT29, SW480, LOVO, HCT116, CACO2 and FHC cells were bought from ATCC (Manassas, VA, USA). Culture medium for HCT116, FHC and CaCO2 was DMEM (Hyclone, Logan, UT) while feed medium for LOVO, SW480 and HT-29 was RPMI-1640 (Hyclone), and both of which were supplemented with penicillin (100 U/ml, Solarbio, Beijing, China), streptomycin (100 µg/ml, Solarbio) and heat-inactivated fetal bovine serum (FBS) (10%, Gibco Laboratories)). CRC cells were cultured at 37 °C under 5% CO_2_ with the confluency above 80%.

To verify how Wnt/β-catenin pathway played in USP6NL-induced SW480 cell growth, after USP6NL overexpression transfection, SW480 cells were treated with 10 μmol/l of XAV939 (S1180, Selleck) and then cultured as mentioned above. To verify the roles of C-myc in regulating USP6NL and SW480 cell growth, after transfection, cells were treated with 100 μmol/l of 10058-F4 (S7153, Selleck).

### Production and transfection of USP6NL overexpression vectors

The primers of human USP6NL gene (NCBI NM_AB449916.1) were: 5′-CGGAATTCATGAATTCAGACCAGGATGTAGC-3′ (forward) and 5′-CGGGATCCTCACAGCAACACTGACTCTTGG-3′ (reverse), which were implanted into pLVX-Puro vector (Clontech). Plasmids (pLVX-Puro-USP6NL), as well as psPAX2 and pMD2G (Addgen) were co-transfected into 293T cells (ATCC) using Lipofectamine™ 2000 (Invitrogen, CA, USA). After 4–6 h, cells were feed in a complete medium, and lentiviruses with USP6NL expression were collected at 48 h and 72 h. 1.5 μg of lentiviruses with USP6NL expression was transfected into SW480 cells using Lipofectamine 2000 reagent. Meanwhile, cells transfected with pLVX-Puro without USP6NL expression were thought as the corresponding control.

### RNA interference

Three siRNAs targeting human USP6NL mRNA (NM_AB449916.1) were designed in Table 1. The siRNAs (20 μmol/l) were transfected into HCT116 and LOVO cells using Lipofectamine^®^ 2000 Reagent (Invitrogen). A non-specific siRNA (siRNA-NC) was used as a negative control.

**Table 1:** siRNA- USP6NL sequences

Label 1	USP6NL sequence:	5′-GGAGCGAGCTGAAATAGTT-3′, start point 39
	siRNA sequence:	5′-CCGGTGGAGCGAGCTGAAATAGTTCTCGAGAACTATTTCAGCTCGCTCCTTTTTG-3′ (forward); 5′-AATTCAAAAAGGAGCGAGCTGAAATAGTTCTCGAGAACTATTTCAGCTCGCTCCA-3′ (reverse)
Label 2	USP6NL sequence:	5′-GCACATTTCGGGACCACAT-3′, start point 449
	siRNA sequence:	5′-CCGGTGCACATTTCGGGACCACATCTCGAGATGTGGTCCCGAAATGTGCTTTTTG-3′ (forward); 5′-AATTCAAAAAGCACATTTCGGGACCACATCTCGAGATGTGGTCCCGAAATGTGCA-3′ (reverse)
Label 3	USP6NL sequence:	5′-GCTTTACTCCTCATGTATA-3′, start point 580
	siRNA sequence:	5′-CCGGTGCTTTACTCCTCATGTATACTCGAGTATACATGAGGAGTAAAGCTTTTTG-3′ (Forward); 5′-AATTCAAAAAGCTTTACTCCTCATGTATACTCGAGTATACATGAGGAGTAAAGCA-3′ (reverse)

### Cell counting kit-8 (CCK-8) assay

Proliferation of HCT116, LOVO and SW480 cells was assessed using CCK8. Briefly, after treatment at 0, 24, 48, 72 h, 3 × 10^3^ cell/well in cultured medium (90 μl/well) were hatched with CCK-8 working solution (10 μl/well) at 37 °C for 1 h. Optical density (OD) value was read by a DNM-9602 microplate reader (PerLong, Beijing, China) at 450 nm.

### Flow cytometry for cell cycle detection

After knockdown of USP6NL, CRC cells were fixed in ethanol, rinsed in PBS, remove RNA using 1 mg/ml of RNase A (R8020-25, Solarbio, Beijing, China), and then incubated with 50 μg/ml of propidium iodide solution (C001-200, 7sea Biotech, Shanghai, China) for 10 min in darkness at 25 °C. DNA content was monitored via flow cytometry (Accuri C6, BD Biosciences, San Jose, CA, USA) with FlowJo v10 (Tree Star, Ashland, OR).

### Dual luciferase report gene assay

USP6NL promoter was amplified with the primers: 5′-CCCTCGAGTATCATTTAATGGGTTCTGGAGGTC-3′ (forward with *Xho*I site as indicated by the underline) and 5′-CCAAGCTTATAGAGTAACTTATCTATTCCAAGATTTC-3′ (reversed, *Hin*dIII site as indicated by the underline), and inserted into pGL3-Enhance to construct pGL3-Enhancer-p USP6NL. SW480 cells (5 × 10^5^ cells/well) on a 6-well plate were cultured at 37 °C overnight for adherent growth, and then transfected with plasmid pGL3-Enhancer-p USP6NL (1.5 μg) and internal control plasmid pRL-TK (0.05 μg) using Lip 2000 (11668-019, Invitrogen) according to a provided instruction. Besides, pGL3-Enhancer was used as a negative control.

6 h later, SW480 cells with USP6NL overexpression were maintain in fresh culture medium with or without 10058-F4 (100 μmol/l) at 37 °C for 24 h. After fully lysis, Dual-Luciferase^®^ Reporter Assay System (E1910, Promega) was used to detect the activity of firefly luciferase in plasmid pGL3-Enhancer and pGL3-Enhancer-p USP6NL, as well as the activity of renilla luciferase in internal control plasmid pRL-TK. The activity of USP6NL promoter was presented as the ratio of firefly/renilla. Each group set 3 wells, and the experiment was repeated 3 times.

### Real-time (RT)-PCR

Total RNA from colorectal tissue, CRC cell lines (HT29, SW480, LOVO, HCT116, CACO2), or human normal colon FHC cells was extracted by Trizol Reagent (1596-026, Invitrogen) and reverse transcribed using cDNA synthesis kit (Fermentas). The primers targeted USP6NL (NCBI NM_AB449916.1) were: 5′-TACTCAGCCTTTCAACTC-3′ (USP6NL-forward) and 5′-GCAAGTACACGTCAAATC-3′ (USP6NL-reverse); Pos: 2389-2604; size: 216 bps; primers targeted glyceraldehyde-3-phosphate dehydrogenase (GAPDH) (NCBI NM_001256799.2) were: 5′-AATCCCATCACCATCTTC-3′ (GAPDH-forward) and 5′-AGGCTGTTG∆∆∆TCATACTTC-3′ (GAPDH-reverse). mRNA levels of USP6NL were determined using SYBR Green PCR Kit (Thermo Fisher) on an ABI7300 system (Applied Biosystem). Amplification condition was: 95 °C, 10 min (95 °C, 15 s; 60 °C, 45 s) × 40; 95 °C, 15 s; 60 °C, 1 min; 95 °C, 15 s; 60 °C, 15 s. USP6NL was normalized by GAPDH and quantified using the 2^−∆∆Ct^ method according to a reported study [15].

### Western blot analysis

Colorectal tissue (20 mg) and CRC cell lines samples were lysed in RIPA (JRDUN, Shanghai, China). Total protein content in supernatant was determined using BCA protein assay kit (PICPI23223, Thermo, MA, USA). Total protein (25 μg) was separated on 15% SDS-PAGE, and electrophoretically pure were sent onto PVDF membranes (Millipore, USA) and incubated with anti-USP6NL antibody (Ab233414, Abcam), anti-β-catenin antibody (#8480, CST), anti-P27 antibody (#3686, CST), anti-Cyclin D1 antibody (#2922, CST), antibody against C-myc (ab32072, Abcam), and antibody against GAPDH (#5174, CST) at 4 °C overnight followed by horseradish peroxidase-conjugated antibodies (Beyotime, Shanghai, China) for 1 h at 25 °C. USP6NL, β-catenin, P27, Cyclin D1 and C-myc were normalized by GAPDH and quantified using ECL system (GE Healthcare/Amersham Biosciences).

### In vitro co-immunoprecipitation (Co-IP) and ubiquitination assay

To study whether USP6NL was associated with β-catenin, 100 μg of total protein in lysis supernatant of HCT116 cells transfected with siUSP6NL was added into protein G-Agarose beads (Roche), and then immune-precipitated with anti-USP6NL (Abcam), anti-β-catenin (Abcam) or control IgG antibody overnight at 4 °C. USP6NL and β-catenin in the immune complex were immunoblotted using anti-USP6NL (NBPI-47264), anti-β-catenin (ab6301, Abcam), respectively according the western blot method. Same amount of protein in each group was reserved for input control.

To study the effect of USP6NL on β-catenin ubiquitination, after fully lysis of HCT116 cells transfected with siUSP6NL, 100 μg of total protein in supernatant was added into Protein A/G PLUS-Agarose (Santa Cruz Biotechnology, sc-2003), and then immune-precipitated with 1 μg of IgG (Santa Cruz Biotechnology, sc-2027) or Anti-β-catenin antibody (ab227499) overnight at 4 °C. Ubiquitination levels of β-catenin in precipitated immune-complexes were separated and quantified using Anti-ubiquitin antibody (ab7780) according the western blot.

### Xenograft model

12 nude mice, 4–6-week-old, were obtained from Shanghai Laboratory Animal Company, and randomly divided into siNC and siUSP6NL groups. Mice in siNC group were subcutaneously injected with HCT116 cells transfected with siNC (7 × 10^6^ cells, 100 μl). Mice in siUSP6NL group were subcutaneously injected with HCT116 cells transfected with siUSP6NL (7 × 10^6^ cells, 100 μl). Then, mice in different groups were separately fed and the bedding was changed regularly. After tumors formation (12 day later), tumor volume (mm^3^) and weight (g) were calculated every 3 days from 12th day to 33th day. Tumor volume were calculated as length × (width^2^/2). We confirmed that the use of mice were abided by the relevant agreements of animal ethics committee in The Affiliated Hospital of Jiangsu University.

### TUNEL reaction

In Situ Cell Death Detection Kit (Roche) was used for detection of cell apoptosis in mouse colorectal tissue. Briefly, 4–7 μm of colorectal tissue section on slides was maintained with 50 μl of TUNEL working solution (11684817910, Roche) at 37 °C for 1 h, and then rinsed twice with PBS. We then dyed the cell nucleus with DBA substrate kit (FL-6001, Long Island, Shanghai, China) followed by hematoxylin (714094, BASO). Individual apoptotic thyroid cells were analyzed using IMS image system (JRDUN, Shanghai, China).

### Statistics and data analysis

Dates were described as mean ± standard error of the mean (SEM), which was calculated by triplicate determinations in each experiment. Comparisons between two groups were assessed by Student’s *t* test with P value < 0.05 being statistically significance.

## Results

### USP6NL was enhanced in CRC

**Fig. 1:**
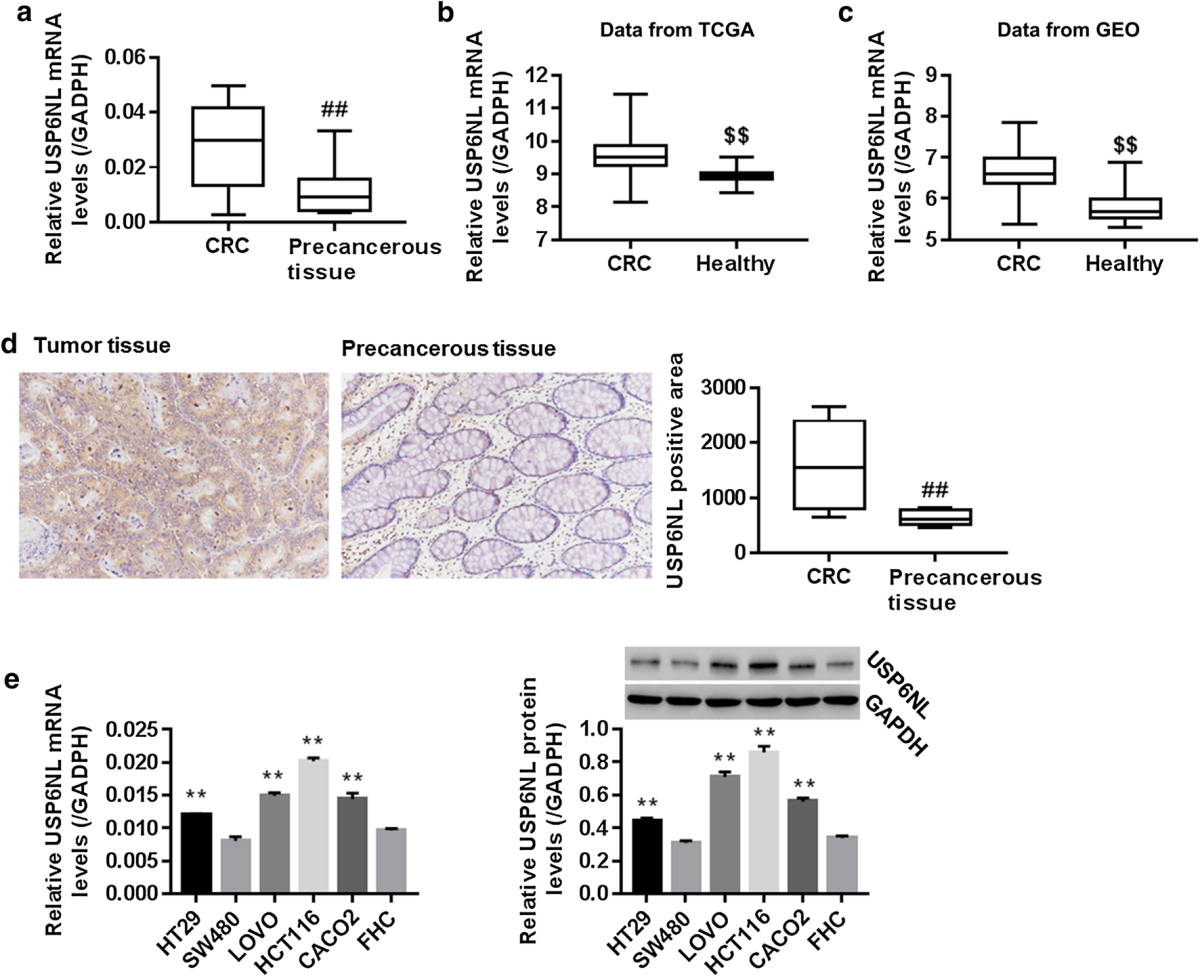
Expression of USP6NL in human CRC. **a** mRNA level of USP6NL in 32 pairs of tumorous colorectal tissues and adjacent non-tumorous tissue, detected using RT-PCT. **b** mRNA level of USP6NL in CRC patients from The Cancer Genome Atlas (TCGA) dataset (n = 260) and corresponding healthy people (n = 41). **c** mRNA level of USP6NL in CRC patients from GEO dataset (n = 70) and corresponding healthy people (n = 12). **d** IHC staining showed that USP6NL was up-regulated in tumor tissue (n = 10) when compared with adjacent-precancerous tissues (n = 5) from CRC patients (original magnification ×200). **e** mRNA and protein levels of USP6NL in five CRC cell lines (HT29, SW480, LOVO, HCT116 and CACO2) and one human normal colon FHC cells were assessed, using RT-PCR and western blot method, respectively. ^##^P < 0.01 vs. precancerous tissue; ^$$^P < 0.01 vs. Healthy people; **P < 0.01 vs. FHC cells

To investigate the involvement of USP6NL in human CRC, USP6NL expression in tumorous and none-tumorous colorectal tissue from CRC patients were detected. USP6NL mRNA expression data in CRC and corresponding healthy people were downloaded in TCGA and GEO database. Our results showed that USP6NL mRNA and protein expression was significantly increased in tumorous colorectal tissue when compared with none-tumorous colorectal tissues (Fig. 1a, d), moreover, data from TCGA and GEO database showed that USP6NL mRNA was significantly increased in CRC patients when compared with corresponding healthy people (Fig. 1b, c), which suggested the participation of USP6NL in human CRC.

Furthermore, mRNA expression of USP6NL in five CRC cell lines (HT29, SW480, LOVO, HCT116 and CACO2) and one human normal colon FHC cells were assessed. Our data suggested that USP6NL was obviously enhanced in CRC cell lines when compared with FHC cells, substantiating the amplification of USP6NL in CRC in vitro (Fig. 1e). Besides, among CRC cell lines, USP6NL was highly expressed in HCT116 and LOVO cells while lowly expressed in SW480 cells. Therefore, we chose HCT116, LOVO and SW480 for the following study.

### Knockdown of USP6NL suppressed CRC cell proliferation and induced cell cycle arrest

**Fig. 2:**
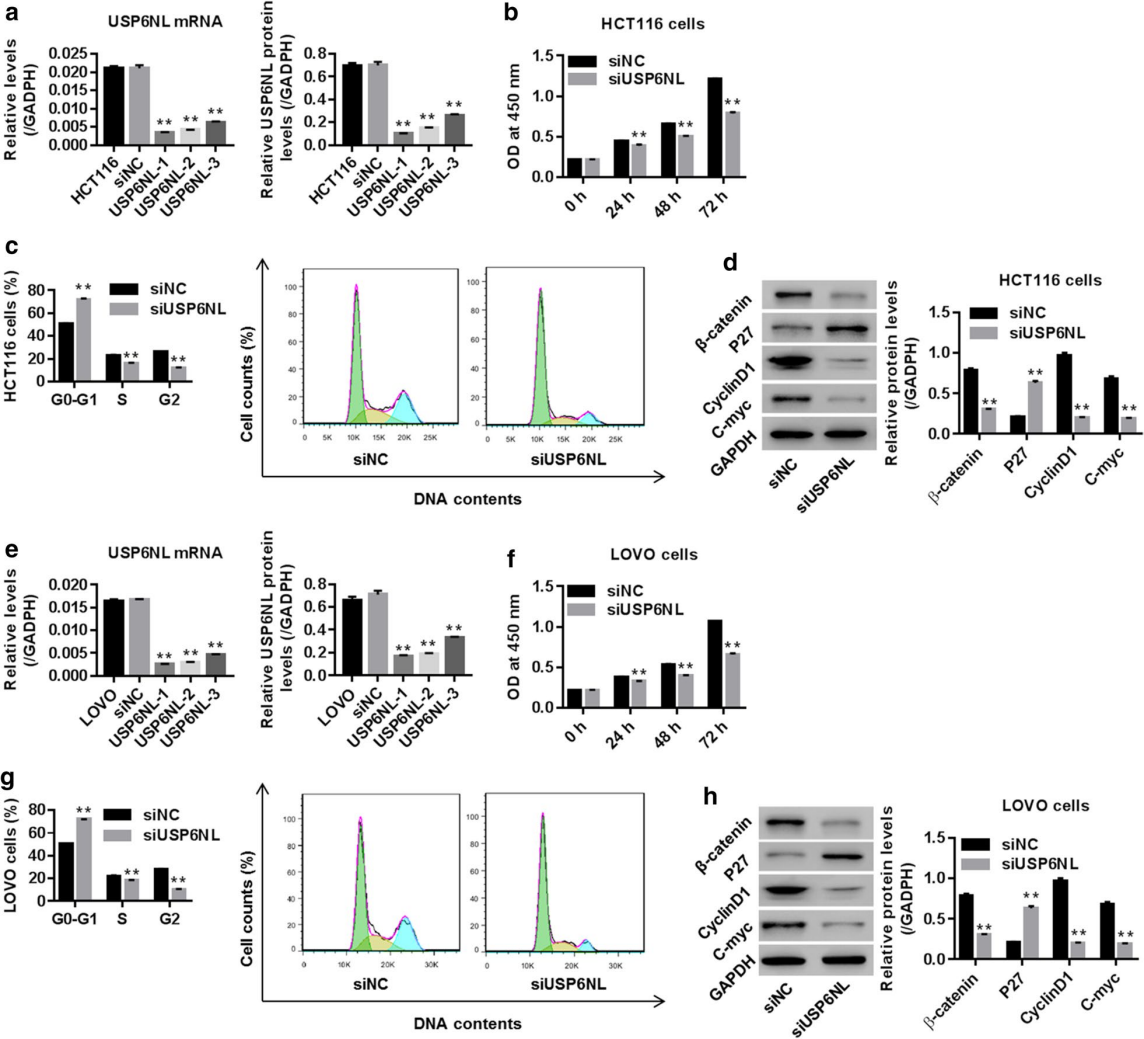
Knockdown of USP6NL inhibited CRC cell growth and prevented Wnt/β-catenin pathway activation. **a**, **e** mRNA and protein levels of USP6NL, assessed by RT-PCR and western blot, respectively, were significantly down-regulated in HCT116 and LOVO cells, suggesting a successful establishment of USP6NL silencing within those two CRC cell lines. **b**, **f** CCK-8 analysis showed that siUSP6NL (siRNA-USP6NL-1) significantly inhibited the proliferation of HCT116 and LOVO cells. **c**, **g** Flow cytometry showed that siUSP6NL obviously enhanced the proportion of HCT116 and LOVO cells in G0–G1 while reduced cell rates in S and G2 phases. **f**, **h** Western blot analysis showed that siUSP6NL remarkably down-regulated β-catenin, Cyclin D1 and C-myc while up-regulated P27 in HCT116 and LOVO cells. **P < 0.01 vs. siNC

HCT116 and LOVO cells were transfected with siRNA-NC (siNC) or siRNA-USP6NL (siUSP6NL-1, siUSP6NL-2 and siUSP6NL-3). Figure 2a, e showed that USP6NL was significantly reduced in siUSP6NL groups (siUSP6NL-1, siUSP6NL-2 and siUSP6NL-3) with the maximum effect being obtained in siUSP6NL-1 when compared with siNC, suggesting the successful establishment of knockdown of USP6NL within those two cell lines. Thus, we chose siUSP6NL-1 for the following study.

The proliferation of CRC cells transfected with siUSP6NL was examined using CCK-8 assay. Figure 2b, f noted that USP6NL silencing time-dependently reduced CRC cell proliferation with the suppressed rates being 13%, 24% and 35% at 24, 48 and 72 h, respectively in HCT116 cells and 13%, 25% and 38% at 24, 48 and 72 h, respectively in LOVO cells. Figure 2c, g showed that knockdown of USP6NL evidently enhanced the percentage of HCT116 and LOVO cells in G0–G1 phase and inhibited the percentage of HCT116 and LOVO cells in S and G2–M phases, demonstrating an obvious cycle arrest in G0–G1 phase in those two cells.

The transition from G0/G1 to S phase is regulated by the P27, Cyclin D1 and C-myc, which are targeted genes of β-catenin (the core element of Wnt/β-catenin pathway) [16]. To investigate the molecular mechanism of USP6NL in the growth inhibition of HCT116 and LOVO cells, protein expression of β-catenin, as well as cell cycle associated P27 (a negative effector of cell cycle progression), Cyclin D1 and C-myc (positive regulator in cell cycle progression) were assessed. Figure 2d, h showed that siUSP6NL dramatically decreased β-catenin, Cyclin D1 and C-myc while increased P27 when compared with siNC within HCT116 and LOVO cells (all P < 0.01).

### Wnt/β-catenin pathway was the mechanism, by which USP6NL regulated CRC cell growth

**Fig. 3:**
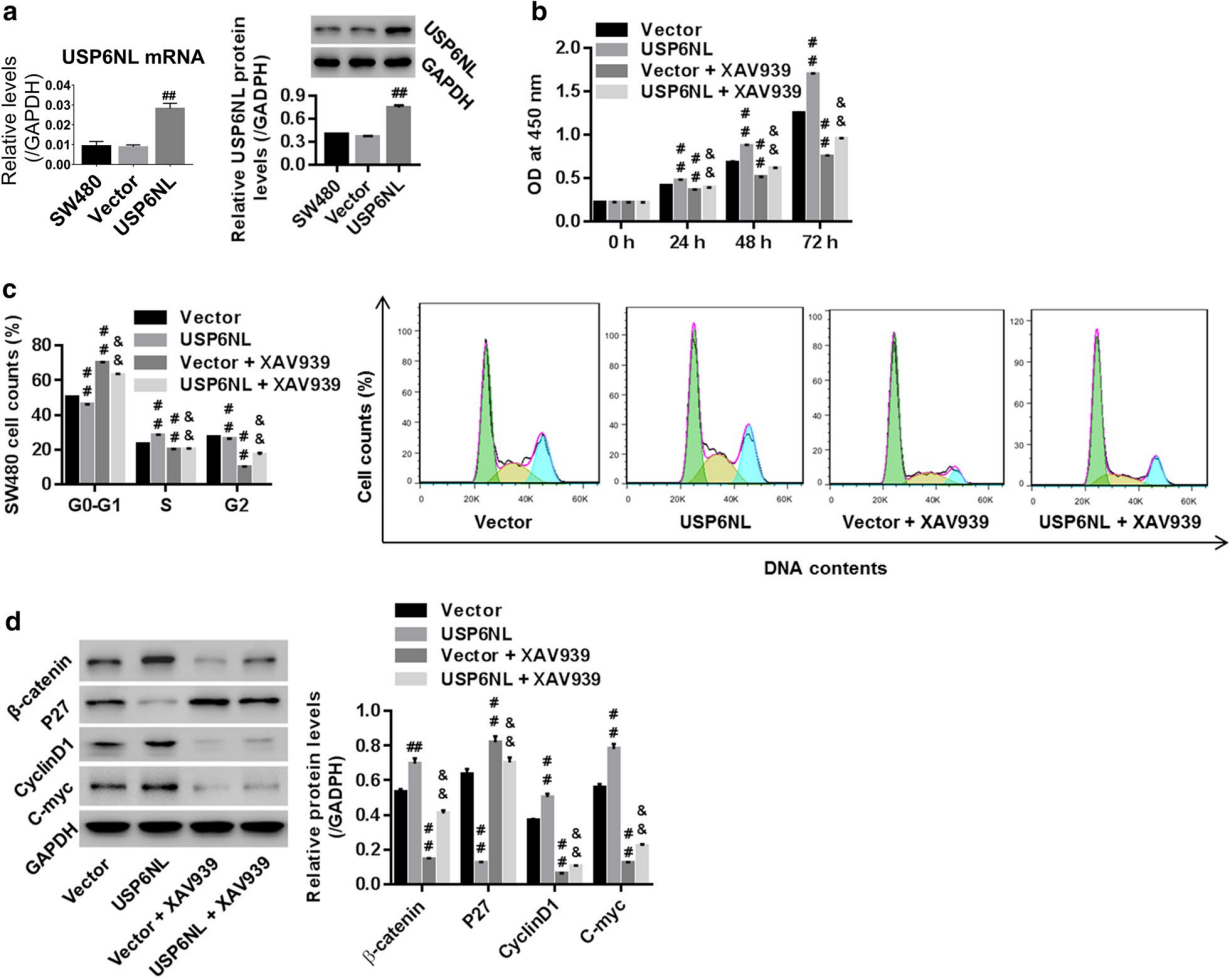
Overexpression of USP6NL promoted CRC cell growth via activating Wnt/β-catenin pathway. **a** Successful establishment of USP6NL overexpression within SW480 cells, assessed by RT-PCR and western blot. USP6NL overexpression transfected SW480 cells were treated with 10 μmol/L XAV939 (Wnt/β-catenin pathway inhibitor), and then **b** cell proliferation. **c** SW480 cell proportion in G0–G1, G2 and S phases, and **d** protein expression of β-catenin, Cyclin D1, C-myc and P27, were assessed using CCK-8 method, flow cytometry, and western blot, respectively. ^##^P < 0.01 vs. vector; ^&&^P < 0.01 vs. vector + XAV939

SW480 cells were transfected with control vector or USP6NL overexpression vector. Figure 3a showed that USP6NL mRNA and protein expression were significantly enhanced in USP6NL group when compared with vector, suggesting the successful establishment of USP6NL overexpression within SW480 cells.

To confirm that Wnt/β-catenin pathway was the mechanism, by which USP6NL regulated cell growth, SW480 cells with USP6NL overexpression were treated with XAV939 (a specific inhibitor for Wnt/β-catenin pathway). Then, cell proliferation, cell cycle, and protein levels of β-catenin, P27, Cyclin D1 and C-myc were assessed. Our data suggested that USP6NL significantly enhanced cell proliferation in a time-depended manner (Fig. 3b), promoted cell cycle progression, by remarkably reducing cell accounts in G0–G1 phase while enhancing cell accounts in S and G2–M phases (Fig. 3c), and up-regulated protein expression of β-catenin, Cyclin D1 and C-myc while down-regulated P27 (Fig. 3d) when compared with vector. On the contrary, cells treated with XAV939 exhibited opposite results. Moreover, the phenotype USP6NL induced was significantly reversed by additional XAV939 treatment. Those results suggested that Wnt/β-catenin pathway is essential for USP6NL to regulate CRC cell growth.

### USP6NL directly interacted with β-catenin and regulated β-catenin ubiquitination

**Fig. 4:**
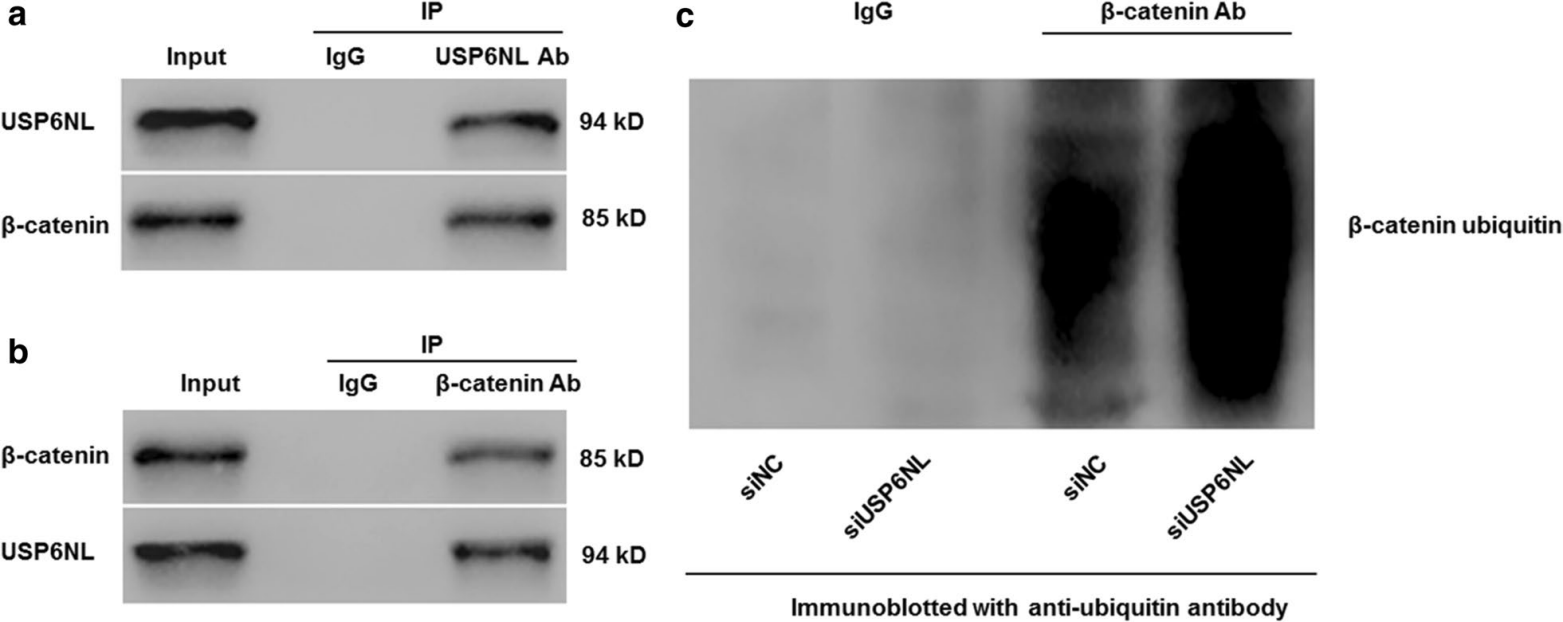
USP6NL was associated with β-catenin, and regulated the ubiquitinylation of β-catenin in human CRC cells. Following the co-immunoprecipitation with **a** anti-USP6NL antibody and **b** anti-β-catenin antibody, the presence of USP6NL and β-catenin in HCT116 cells transfected with siUSP6NL was measured by Western blot. **c** The presence of β-catenin in siUSP6NL transfected HCT116 cells was immunoprecipitated and immunoblotted with β-catenin antibodies or anti-ubiquitin antibodies

To explore how USP6NL regulates β-catenin, we immunoprecipitated USP6NL (or β-catenin) from HCT116 cells, and then β-catenin (or USP6NL) was immunoblotted to analyzed the physical interaction between USP6NL and β-catenin. We found that USP6NL significantly bound β-catenin within CRC cells (Fig. 4a, b).

Without Wnt, β-catenin can be degraded through the ubiquitination/proteasome pathway [17]. Presently, we examined if USP6NL controls β-catenin ubiquitination in HCT116 cells transfected with siUSP6NL. Our data suggested that the levels of ubiquitinated β-catenin were significantly enhanced in siUSP6NL when compared with siNC, (Fig. 4c), demonstrating that USP6NL may be a deubiquitinase that prevents β-catenin ubiquitination.

### C-myc was the mechanism, by which USP6NL regulated CRC cell growth

**Fig. 5:**
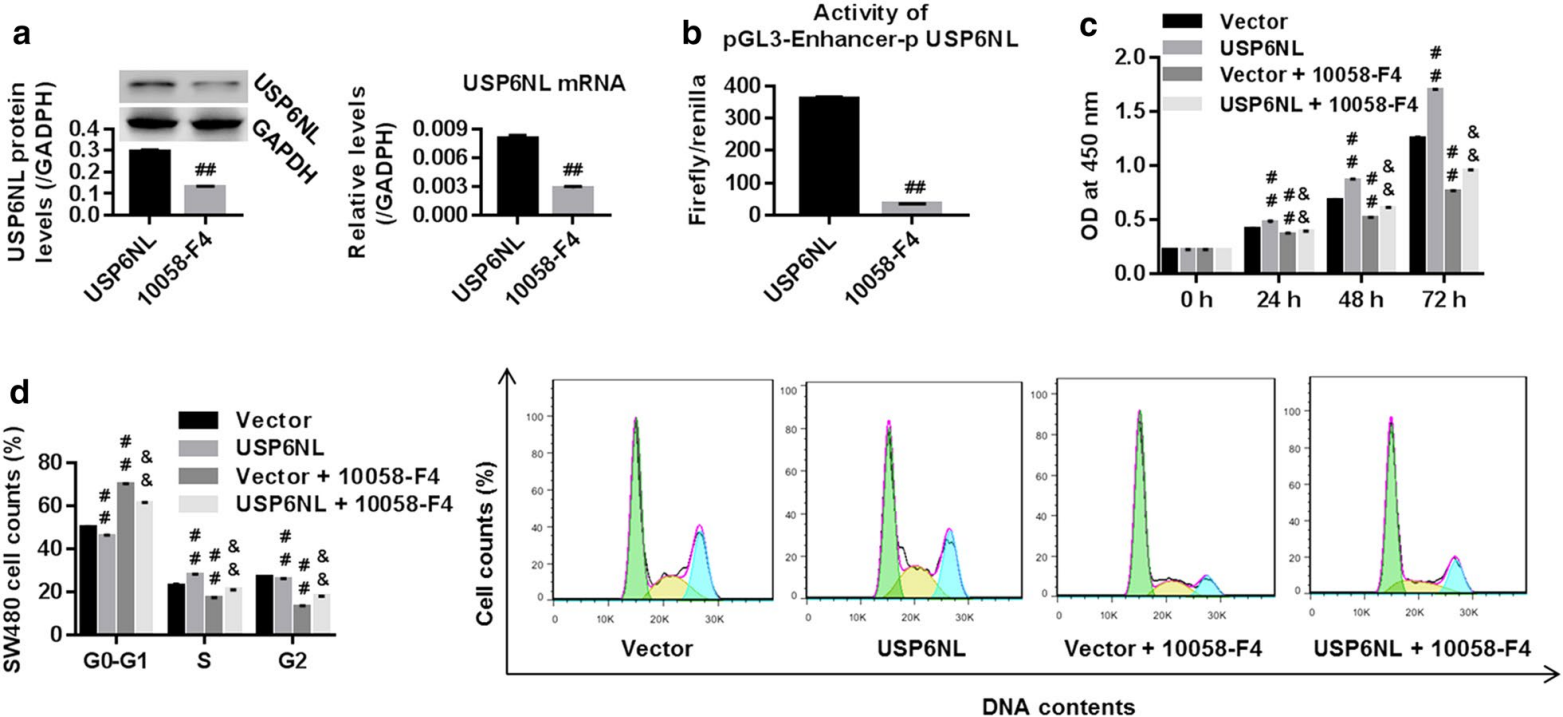
USP6NL acted on C-myc to regulate CRC cell growth. USP6NL overexpression transfected SW480 cells were treated with of a C-myc inhibitor 10058-F4 (100 μmol/l). Decreased **a** mRNA and protein levels and **b** reduced activity of USP6NL promoter (assessed using Dual luciferase report gene assay) in USP6NL overexpression transfected SW480 cells, suggested an inhibitory effect of 10058-F4 on USP6NL expression. **c** Cell proliferation and **d** cell cycle were assessed using CCK-8 method and flow cytometry, respectively. ^##^P < 0.01 vs. vector; ^&&^P < 0.01 vs. vector + 10058-F4

To confirm that C-myc was the mechanism, by which USP6NL regulated cell growth, control vector/USP6NL overexpression transfected SW480 cells were treated with 100 μmol/l of 10058-F4 (a specific C-myc inhibitor). Our data suggested that 10058-F4 significantly reduced USP6NL mRNA and protein levels (Fig. 5a), and suppressed the activity of USP6NL promoter (Fig. 5b) when compared with vector, demonstrating the inhibited effect of 10058-F4 on USP6NL expression. Besides, in comparison to Vector/USP6NL treatment, 10058-F4 time-dependently inhibited cell proliferation (Fig. 5c), and induced a server cycle arrest in G0–G1 phase through enhancing cell counts in G0–G1 phase while inhibiting cell counts in S and G2–M phases (Fig. 5d). Given the roles of USP6NL in regulating C-myc and cell growth, we can conclude that USP6NL acted on C-my to regulate CRC cell growth.

### Knockdown of USP6NL suppressed tumorigenicity of HCT116 cells in nude mice

**Fig. 6:**
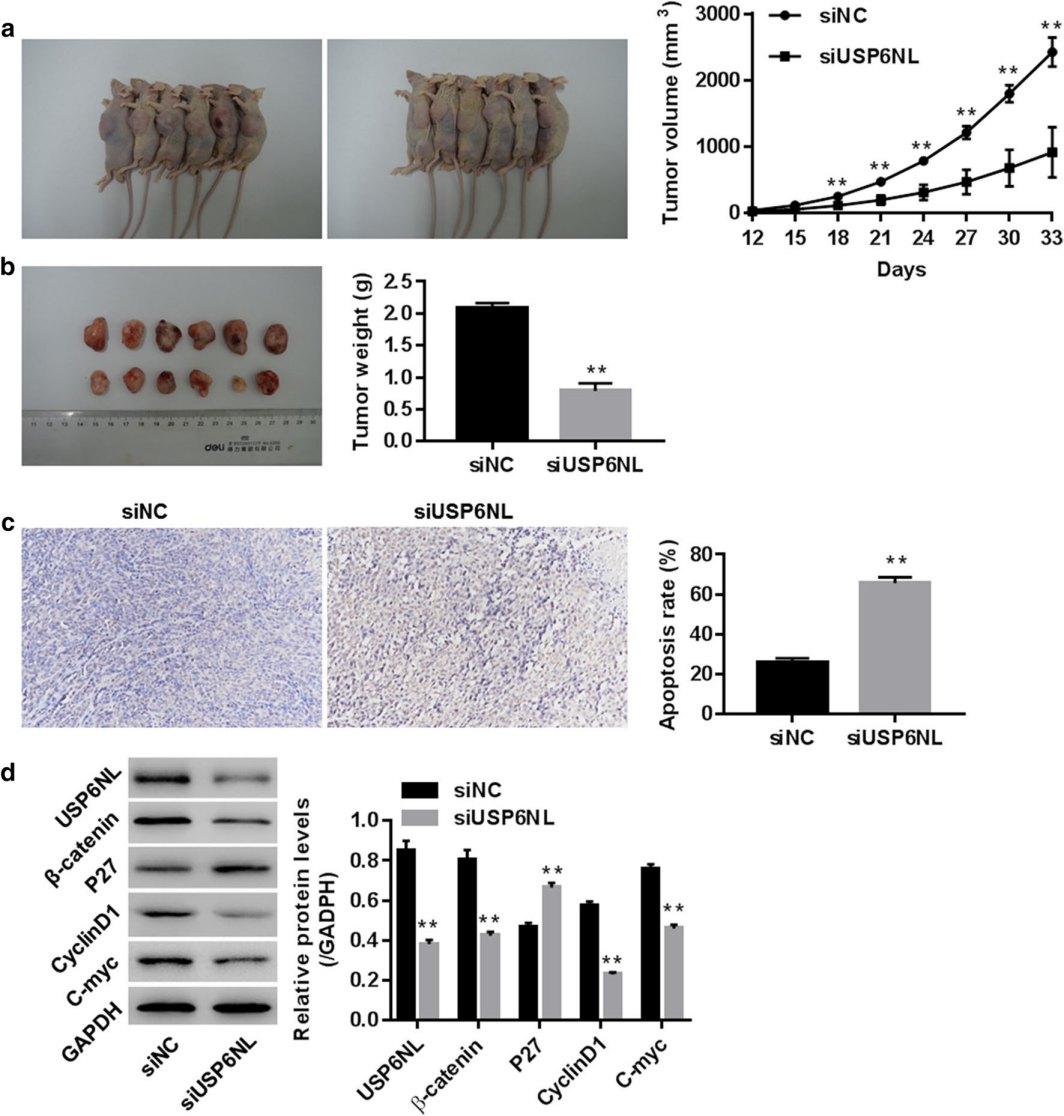
Knockdown of USP6NL inhibited tumorigenicity of HCT116 cells in a Xenograft model. Nude mice were subcutaneously injected with siNC/siUSP6NL transfected HCT116 cells (7 × 10^7^ cells, 100 μl) (n = 6 in each group). **a** Tumor formation of mice and tumor volume (mm^3^) from 12th day to 33th day; **b** tumor weight (g) on 33th day; **c** apoptosis analysis in tumor using TUNEL technology (×200; **d** protein levels of USP6NL, β-catenin, Cyclin D1, C-myc and P27 in tumors; **P < 0.01 vs. siNC

After injection the nude mice with HCT116 cells transfected with siNC or siUSP6NL, tumor volume and weight were examined for 22 days, tumor apoptosis was measured using TUNEL reaction. Our data showed that siUSP6NL significantly inhibited tumor volume and tumor weight (Fig. 6a, b, all P < 0.01), promoted apoptosis in tumor tissue (Fig. 6c), reduced USP6NL, β-catenin, Cyclin D1 and C-myc while increased P27 when compared with siNC group (Fig. 6d), suggesting the involvement of USP6NL and its potential association with Wnt/β-catenin pathway and C-myc in CRC in vivo.

## Discussion

Ubiquitin-specific proteases (USPs), acting as deubiquitinating enzymes (DUBs), is emerging as the master regulator of tumor [10, 18–21]. In recent years, more and more evidence that the USPs family in oral malignant tumor, liver cancer, gastric cancer, breast cancer, colorectal cancer, and other abnormal expression in tumor [22]. In recent years, studies have found that USPs members USP4, USP7 and USP47 have been identified as β-catenin-specific deubiquitinylating enzyme [10–12]. As a member of the USP family, USP6NL plays an important role in the physiological process of the body [23, 24], and is closely related to Wnt/β-catenin signaling. Therefore, we propose whether USP6NL is involved in colorectal cancer cell proliferation and metastasis through Wnt/β-catenin signaling. At present, there are few studies on the role of USP6NL in CRC. Whether and how USP6NL acted on Wnt/β-catenin signaling pathway in CRC remained extremely poor requires further exploration.

Previous studies have shown that the cell cycle of tumor cells is significantly different from that of normal tissue cells, and its S and G2–M phase is significantly higher than that of ordinary cells, while the percentage of G0–G1 phase representing cell division stagnation is lower [25]. In this study, we found that knockdown of USP6NL significantly enhanced the percentage of CRC cells in the G0–G1 phase and inhibited the percentage of CRC cells in the S and G2–M phases. Therefore, we proved that the overexpression of USP6NL in CRC cells enhanced cell proliferation and promoted cell cycle progression from G0/G1 phase to S phase. Knockdown of USP6NL has the opposite effect, inhibiting CRC cell proliferation and inducing cell cycle arrest at G0/G1 phase. The transition from G0/G1 to S is mediated by P27, Cyclin D1, and c-myc, which are target genes for β-catenin (the core component of the Wnt/β-catenin pathway) [26].

We then investigated whether and how USP6NL acts on Wnt/β-catenin to regulate CRC cell growth. By treating USP6NL overexpressing CRC cells with XAV939, a specific inhibitor of the Wnt/β-catenin pathway, the results suggest that CRC cell proliferation, G1/S cell cycle progression, and β-catenin Cyclin D1 and C-myc are promoted. Enhanced expression, while a decrease in P27 induced by overexpression of USP6NL, was significantly reversed, suggesting that activation of the Wnt/β-catenin pathway is a mechanism by which USP6NL is carcinogenic to CRC in vitro. Our data suggested that 10058-F4 (C-myc inhibitor) significantly reduced USP6NL mRNA and protein levels, and suppressed the activity of USP6NL promoter when compared with vector, demonstrating the inhibited effect of 10058-F4 on USP6NL expression.

Furthermore, our data suggest that knockdown of USP6NL increases ubiquitination of β-catenin, suggesting that USP6NL may act as a deubiquitinating enzyme that regulates β-catenin accumulation during this process. In addition, 10058-F4 downregulated USP6NL, inhibited CRC cell proliferation and induced cell cycle arrest, demonstrating that USP6NL//β-catenin/C-myc may have a feedback loop in regulating CRC cell growth.

Our study reveals the new highlight is that USP6NL activated Wnt/β-catenin pathway probably by the inhibition of β-catenin ubiquitination, revealing other pathways that have not been prioritized, including the Wnt/β-catenin pathway, and has filled a gap in the mechanism of USP6NL’s impact on CRC.

The tumor growth rate and size of the mouse model siRNA-USP6NL group were significantly lower than that of the siRNA-NC group, which provided a strong basis for the purpose of this study, and provided a possibility for the subsequent human targeted therapy research of USP6NL.

However, there are some limitations in our study. The specific mechanism of how USP6NL may activate the Wnt/beta-catenin pathway by inhibiting ubiquitination of beta-catenin is still not clear enough. The treatment of USP6NL inhibitor has not been added in animal experiments, which brings it closer to clinical trials and provides more background information for later experiments.

**Fig. 7:**
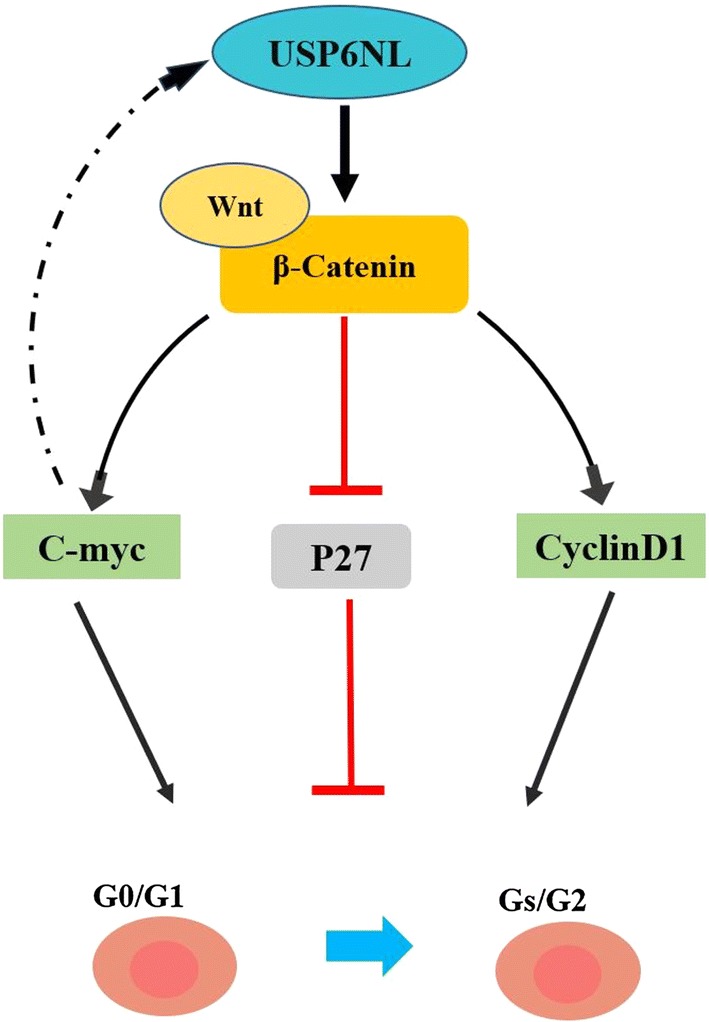
The relationship between USP6NL and the pathway

In conclusion, USP6NL is closely related to the production of CRC, and its mechanism may be affected by intervention in the Wnt/β-catenin signaling pathway. Our data provide close evidence. However, the exact mechanism by which USP6NL affects the Wnt/β-catenin signaling pathway is unclear and needs further study. Taken together, our study not only indicates that USP6NL is up-regulated in CRC tissues, but also emphasizes its role in regulating CRC cell proliferation and metastasis in vivo and in vitro. In addition, we also found that USP6NL regulates the potential mechanism of CRC cell proliferation and metastasis by participating in the Wnt/β-catenin signaling pathway (Fig. 7). This may make it possible for USP6NL to treat CRC with molecularly targeted drugs.

## In conclusion

USP6NL was an oncogene in the tumorigenesis of CRC both in vitro and in vivo, and activation of Wnt/β-catenin pathway was the underlying mechanism. USP6NL activated Wnt/β-catenin pathway probably by the inhibition of β-catenin ubiquitination. Our research elucidated that targeting USP6NL may represent a novel therapeutic target in CRC treatment.
